# Missing Care: the Initial Impact of the COVID-19 Pandemic on CKD Care Delivery

**DOI:** 10.1007/s11606-022-07805-w

**Published:** 2022-09-26

**Authors:** Clarissa J. Diamantidis, David J. Cook, Stephan Dunning, Cyd Kristoff Redelosa, Martin Francis D. Bartolome, Roland Albert A. Romero, Joseph A. Vassalotti

**Affiliations:** 1 Duke University School of Medicine, Durham, NC USA; 2 Optum Labs, Minnetonka, MN USA; 3 National Kidney Foundation, New York, NY USA; 4 Icahn School of Medicine at Mount Sinai, New York, NY USA

**Keywords:** chronic kidney disease, COVID-19, epidemiology

## Abstract

**Background:**

Chronic kidney disease (CKD) is a common condition with adverse health outcomes addressable by early disease management. The impact of the COVID-19 pandemic on care utilization for the CKD population is unknown.

**Objective:**

To examine pandemic CKD care and identify factors associated with a high care deficit.

**Design:**

Retrospective observational study

**Participants:**

248,898 insured individuals (95% Medicare Advantage, 5% commercial) with stage G3–G4 CKD in 2018

**Main Measures:**

Predicted (based on the pre-pandemic period of January 1, 2019–February 28, 2020) to observed per-member monthly face-to-face and telehealth encounters, laboratory testing, and proportion of days covered (PDC) for medications, evaluated during the early (March 1, 2020–June 30, 2020), pre-vaccine (July 1, 2020–December 31, 2020), and late (January 2021–August 2021) periods and overall.

**Key Results:**

In-person encounters fell by 24.1% during the pandemic overall; this was mitigated by a 14.2% increase in telehealth encounters, resulting in a cumulative observed utilization deficit of 10% relative to predicted. These reductions were greatest in the early pandemic period, with a 19.8% cumulative deficit. PDC progressively decreased during the pandemic (range 9–20% overall reduction), with the greatest reductions in hypertension and diabetes medicines. CKD laboratory monitoring was also reduced (range 11.8–43.3%). Individuals of younger age (OR 1.63, 95% CI 1.16, 2.28), with commercial insurance (1.43, 95% CI 1.25, 1.63), residing in the Southern US (OR 1.17, 95% CI 1.14, 1.21), and with stage G4 CKD (OR 1.21, 95% CI 1.17, 1.26) had greater odds of a higher care deficit overall.

**Conclusions:**

The early COVID-19 pandemic resulted in a marked decline of healthcare services for individuals with CKD, with an incomplete recovery during the later pandemic. Increased telehealth use partially compensated for this deficit. The downstream impact of CKD care reduction on health outcomes requires further study, as does evaluation of effective care delivery models for this population.

**Supplementary Information:**

The online version contains supplementary material available at 10.1007/s11606-022-07805-w.

## INTRODUCTION

Chronic kidney disease (CKD) is a common and costly condition, affecting approximately 15% of US adults (roughly 37 million Americans).^1,2^ In 2018, Medicare expenditures for patients with documented CKD (non-dialysis) exceeded $81 billion, almost a quarter of all US Medicare fee-for-service beneficiary spending.^3^ Despite the prevalence and expenditures for CKD, outcomes remain poor. Individuals with CKD are at a higher risk of heart disease, stroke, and premature death.^4,5^ Early identification and treatment is critical to improving CKD outcomes, although barriers to optimal CKD care exist: (1) low patient and clinician diagnoses, (2) complexities in navigating health systems by individuals with complex health needs, (3) inequities in diagnoses and treatments, (4) care fragmentation, and (5) payment models heavily skewed to late-stage disease and dialysis. These synergistically contribute to the vulnerability of the US CKD population.

The COVID-19 pandemic has dramatically influenced care delivery. Social distancing, facility closures, and competing personal demands have led to dramatic reductions in overall healthcare utilization.^6^ Significant modifications in care delivery, particularly in the early pandemic period, have been noted across multiple conditions,^7,8^ settings,^9–11^ and subgroups.^12–14^ For individuals with CKD, the pandemic has also had significant adverse health effects.^15,16^ Individuals with CKD are at higher risk of more severe illness and death from the SARS-CoV-2 virus.^17^ Furthermore, acute kidney injury is a common outcome associated with SARS-CoV-2 hospitalization and a risk factor for CKD progression.^18^ Despite mounting evidence of the adverse clinical effects of SARS-CoV-2 infection on kidney health, little is known regarding patterns of care disruptions in the CKD community. Herein, we sought to characterize disruptions in CKD care using a national cohort of individuals with non-dialysis CKD.

## METHODS

### Study Population

We performed a retrospective study of 5,236,344 de-identified individuals continuously enrolled in commercial and Medicare Advantage (MA) health plans through UnitedHealth Group from January 2018–August 2021. Evidence of CKD was determined using de-identified claims and/or lab results data and defined by a member having either ≥ 2 CKD claims (based on ICD-10 N18 codes) or two eGFR results < 60 ml/min/1.73 m^2^ at least 90 days apart in 2018. Estimated GFR was calculated from serum creatinine values using the 2021 CKD-EPI equation.^19^ CKD stage was determined based on the most recent eGFR (if present) or ICD-10 code in the baseline period. Individuals with stage G5 CKD, end-stage kidney disease (ESKD)–related claims, or eGFR < 15 ml/min/1.73 m^2^ between January 2018 and August 2021 were excluded, leaving a final sample of 248,898 individuals with CKD G3–G4 (eGFR 30–59 ml/min/1.73 m^2^) for analysis (Figure S1).

The COVID-19 pandemic timeframe of March 2020–August 2021 was divided into three periods: early (March 1, 2020–June 30, 2020), pre-vaccine (July 1, 2020–Dec 31, 2020), and late (January 1, 2021–August 31, 2021) periods. In a population-level analysis, we compared monthly healthcare utilization (office-based and telehealth visits), laboratory test monitoring, and medications (for those with pharmacy coverage) in the pre-pandemic period (January 1, 2019–February 29, 2020) to observed utilization, test monitoring, and medication use in both overall and each respective pandemic period. We then performed a member-level analysis to identify subgroups with high care deficits during the pandemic.

### Healthcare Utilization

Healthcare utilization in the cohort was measured to quantify reductions in care before, and during, the pandemic. Reductions were calculated by determining the average monthly number of outpatient consultations during the pre-pandemic baseline period from January 2019 to February 2020 and comparing predicted monthly utilization to observed average utilization from March 2020 to August 2021. Claims for face-to-face consultations were defined as physician services for office or other outpatient services (using CPT codes 99201-99215 and 99241-99245) with office as the place of service (AMA code 11). Telehealth claims were defined using the same CPT codes as face-to-face claims with the addition of procedure modifiers (95, GT, GQ). E-visit and audio-only encounter procedure codes (CPT 99421–99423, 99441–99443, or HCPCS codes G2061–G2062), which are non-face-to-face patient-initiated communications through patient portals, were excluded to consistently compare face-to-face consultations and telehealth services.

In a member-level analysis, we evaluated sociodemographic (age group, sex, race, insurance type, urban-suburban-rural (USR) classification, geographic region) and clinical (CKD stage) factors associated with high care deficit versus no high care deficit using multivariable logistic regression. High care deficit during the pandemic was defined as the upper 25^th^ percentile of drop-off in face-to-face utilization minus the increase in telehealth utilization from pre-pandemic to the overall pandemic period.

### Laboratory Testing Procedures

We evaluated rates of common laboratory test procedures for monitoring CKD for the pre-pandemic and pandemic periods for the CKD cohort (e.g., comprehensive metabolic panel, complete blood count, urinary albumin-creatinine ratio). Reductions in utilization for lab procedures were determined by calculating the relative difference between the average number of laboratory test claims per member per month (PMPM) during pandemic and pre-pandemic periods.

### Prescription Medication Use

Prescription medication use was ascertained among the 93.4% (228,952) of the CKD cohort with prescription drug coverage. To evaluate changes in medication use, we estimated the proportion of days covered (PDC) for each member. PDC was based on the day’s supply acquired from claims data and the fill date as the starting date for that supply. Only drugs used by ≥ 5% of the cohort were included. Individual medication data was aggregated into drug categories according to the American Hospital Formulary Service Pharmacologic-Therapeutic Classification System.

### Statistical Analysis

Study cohort characteristics were summarized using mean and standard deviation for continuous variables and frequency and percentage for categorical variables. To examine factors independently associated with a high care deficit in the pandemic period, we performed a multivariable logistic regression with the dependent variable high care deficit (versus no high care deficit), adjusted for age group, sex, race, insurance type, urban-suburban-rural (USR) classification,^20^ geographic region, and CKD stage. Calculations were performed using Python version 3.3 and R-version (4.1.2).

## RESULTS

Among the 5,236,344 individuals (3.2M MA, 2.0M Commercial) with continuous enrollment from January 2018 to August 2021, 248,898 were included in the analytic cohort. In the CKD cohort, mean age was 79.1 years, 58.5% were female, and 16.6% were classified as Black. The majority of insurance was Medicare Advantage (95.8%). Baseline stage G3 CKD was noted in 94.1% of the cohort, with 5.9% stage G4 (Table 1).

**Table 1:** Study Population Characteristics

Characteristic	Value (*n* = 248,898)
Age	
Mean (years)	79.1
Standard deviation (years)	8.7
Sex	
Male (%)	41.5
Race	
Asian (%)	1.4
Black (%)	16.6
Hispanic (%)	1.8
Native American (%)	0.12
Others/unknown (%)	9.8
White (%)	70.3
Insurance type	
Medicare Advantage (%)	95.4
Private commercial plan (%)	4.6
USR class	
Urban (%)	24.3
Suburban (%)	31.2
Rural (%)	42.9
Geographic region	
Northeast (%)	14.0
Midwest (%)	15.0
South (%)	53.1
West (%)	17.8
Other (%)	0.11
Chronic kidney disease stage in 2018	
Stage 3 (%)	94.1
Stage 4 (%)	5.9

*USR* urban-suburban-rural

### Healthcare Utilization

Clinician encounters during the pre-pandemic period averaged 1576 visits per month per 1000 patients. For the overall pandemic period (March 2020–August 2021), face-to-face encounters decreased by 24.2% (Table 2). Telehealth services increased to 14.2% of all visits during the pandemic, resulting in an overall average reduction in monthly encounters of 10% for the CKD population. Both reduction in healthcare utilization and increase in telehealth services were most profound in the early pandemic (March 2020–June 2020), resulting in a 19.8% average monthly care reduction (Table S1a). Over time, increases in face-to-face encounters, and decreasing telehealth services, resulted in an approximately 7% reduction in monthly encounters in both the pre-vaccine (July 2020–December 2020) and late (January 2021–August 2021) periods.

**Table 2:** Overall Pandemic Face-to-Face and Telehealth Utilization

Consultations	Per 1000 persons per month
Baseline for the entire cohort	802.42
Reduction, F2F only (%)	194.01 (24.18)
Reduction, F2F + TH (%)	80.10 (9.98)
Telehealth (%)	113.91 (14.20)

*Note: Results are based on chronic kidney disease stages G3 and G4 member cohort (n = 248,898)*

*F2F* face to face, *TH* telehealth

**Figure 1:**
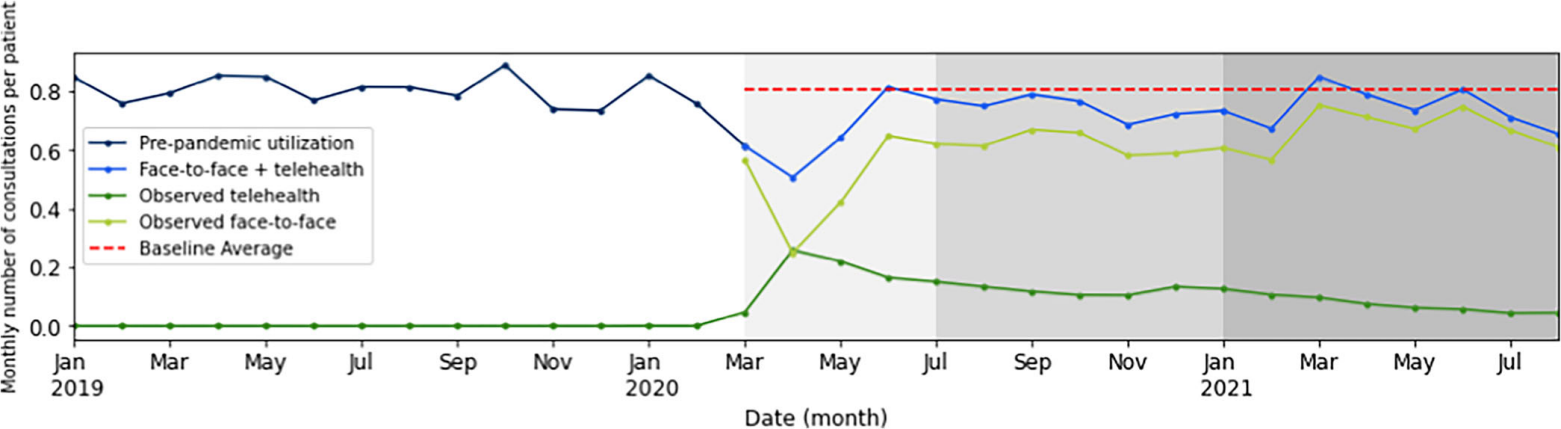
Healthcare utilization in the chronic kidney disease cohort during the COVID-19 pandemic: observed face-to-face office visits and/or telehealth visits per person per month compared to the baseline average. The shaded regions indicate the three COVID sub-periods: early COVID (lightest), pre-vaccine, and late (darkest).

Monthly number consultations per patient (Fig. 1) showed a dramatic reduction in face-to-face visits during March–April 2020, at their nadir ~ 20% below mean 2019 levels. Telehealth visits rose in April 2020 but declined in May and June 2020. By mid-June 2020, face-to-face and telehealth visits in combination approached average pre-pandemic visit levels, followed by temporary declines in late 2020 and July–August 2021.

### Laboratory Testing Procedures

Laboratory testing utilization fell sharply across the most prevalent pre-pandemic laboratory procedures (Table S2a, Table S2b). Overall pandemic PMPM reductions ranged from 11.8 to 43.3% and were most profound during the early pandemic period, with reductions of 25 to 50.2%. Common CKD blood testing procedures such as metabolic panels and complete blood counts followed this overall trend, remaining slightly below pre-pandemic levels in the late pandemic period. In contrast, quantitative urine albumin-creatinine ratio (uACR) testing fell by 31.6% in the early pandemic and remained 17.4% below pre-pandemic levels in the late pandemic period.

### Prescription Medication Use

Relative to pre-pandemic levels, prescription medication use decreased by 9 to 20% PDC (Table S3). These classes included a large proportion of antihypertensive medications and diabetes medications. Notably, PDC reductions increased in each successive pandemic period. For example, beta-adrenergic blockers, the most prevalent drug class, saw a PDC reduction ranging from a nadir of 6% in the early pandemic period to a peak of 13% in the late pandemic period. The most prominent reduction in PDC of 26% was noted for thiazide diuretics in the late pandemic period.

### Factors Associated with Care Deficits

Median PMPM difference in outpatient physician visits before and during the pandemic was 0.16 [IQR − 0.23, 0.60], indicating a reduction of 0.16 visits monthly. Due to the low pre-pandemic use of telehealth, and only transient rise in telehealth during the pandemic period, the median overall difference in telehealth visits before and during the pandemic was 0 [IQR 0, 0].

Therefore, 62,764 individuals were classified as having a high care deficit (PMPM difference ≥ 0.60) and 62,512 were classified as a low care deficit (PMPM differenc ≤ −0.23). Individuals ≥ 75 years, females, and White individuals comprised a larger proportion of individuals in the high care deficit group compared with the low care deficit group (Table 3).

**Table 3:** Demographics of Individuals with High and Low Care Deficits During the COVID-19 Pandemic

	High care deficit	Low care deficit
	(*n* = 60,535)	(*n* = 60,130)
Age group		
0–17	0 (0%)	5 (0.01%)
18–44	79 (0.13%)	143 (0.24%)
45–54	417 (0.69%)	499 (0.83%)
55–64	2431 (4.02%)	2405 (4.00%)
65–74	14,410 (23.8%)	15,318 (25.47%)
75–84	26,854 (44.36%)	25,410 (42.26%)
85+	16,344 (27.00%)	16,350 (27.19%)
Sex		
Male	25,804 (42.63%)	24,732 (41.13%)
Race		
Asian	728 (1.20%)	882 (1.47%)
Black	9072 (14.99%)	10,344 (17.20%)
Hispanic	1266 (2.09%)	948 (1.58%)
Native American	74 (0.12%)	58 (0.1%)
Others/unknown	920 (1.52%)	1113 (1.85%)
White	44,811 (74.02%)	40,976 (68.15%)
Insurance type		
Medicare Advantage	58,304 (96.31%)	56,485 (93.94%)
Private commercial plan	2231 (3.69%)	3645 (6.06%)
USR class		
Urban	14,436 (23.85%)	14,576 (24.24%)
Suburban	19,872 (32.83%)	18,541 (30.83%)
Rural	25,047 (41.38%)	26,278 (43.7%)
Geographic region		
Northeast	8187 (13.52%)	8679 (14.43%)
Midwest	6849 (11.31%)	10,319 (17.16%)
South	34,289 (56.64%)	30,875 (51.35%)
West	11,150 (18.42%)	10,197 (16.96%)
Other	60 (0.10%)	60 (0.10%)
Chronic kidney disease stage in 2018		
Stage 3	56,532 (93.39%)	57,026 (94.84%)
Stage 4	4003 (6.61%)	3104 (5.16%)

After adjustment, individuals in younger age categories (versus 65–74 years), male sex, with commercial (versus MA) insurance, those residing in the south and west (versus northeast), and individuals with stage CKD (versus stage G3) were more likely to experience a high care deficit during the pandemic (Table 4). Individuals ≥ 85 years of age, those of Asian, Black, or other race/ethnicity, and those residing in rural or suburban locations (versus urban) had lower odds of a high care deficit.

**Table 4:** Factors Associated with High Care Deficit During the COVID-19 Pandemic

	Variable	Odds ratio [LCL, UCL]
Age group	65–74 (reference)	N/A
	18–44	1.63 [1.16, 2.28]
	45–54	1.60[1.39,1.84]
	55–64	1.56 [1.47,1.65]
	75–84	1.05 [1.02, 1.07]
	85+	0.96 [0.94, 0.99]
Sex	Male	1.08 [1.06, 1.10]
Race	White (reference)	N/A
	Asian	0.72 [0.66, 0.78]
	Black	0.78 [0.76, 0.80]
	Hispanic	1.04 [0.97, 1.15]
	Native American	1.09 [0.84, 1.40]
	Other	0.72 [0.67, 0.78]
Insurance type	Medicare (reference)	N/A
	Commercial	1.43 [1.25, 1.63]
USR class	Urban (reference)	N/A
	Rural	0.89 [0.86, 0.91]
	Suburban	0.94 [0.92, 0.96]
Geographic region	Northeast	N/A
	Midwest	0.78 [0.75, 0.81]
	South	1.17 [1.14, 1.21]
	West	1.09 [1.05, 1.12]
Chronic kidney disease stage	3 (reference)	N/A
	4	1.21 [1.17, 1.26]

*USR* urban-suburban-rural

## DISCUSSION

In over 248,000 individuals with stage G3 or G4 CKD, we found a dramatic reduction in overall healthcare utilization during the early months of the COVID-19 pandemic with an incomplete rebound. Despite an early increase in telehealth, its utilization only partially compensated for the reduction in face-to-face encounters, leaving an early care deficit of approximately 20% per 1000 persons per month compared with pre-pandemic, and a 10% care deficit during the pandemic overall. We also observed decreases in medication refills for CKD and its risk factors, which became more pronounced as the pandemic proceeded. There were also reductions in CKD lab monitoring, particularly for uACR testing.

It is important to note that reporting results for 20 months of the pandemic as a whole may under-represent the impact of a care deficit. In the first 4 months of the pandemic (early period), care for CKD patients was reduced by 20%. However, service delivery progressively recovered between May of 2020 and August of 2021. By the latest study period, Jan–Aug 2021 care delivery recovered to 7.3% of what was expected; notably, this late period was eight months, while the most dramatic, early period was four months. As such when looking at the pandemic as a whole, the results reported disproportionately represent the latest period and under-represent the early period. While averaging the care deficit over the entire pandemic is technically correct to represent care overall, it may be that the clinical impact of a shorter severe deficit early could be greater than a longer mild deficit in care in the middle and late phases of the pandemic.

These findings indicate a considerable reduction in CKD care management during the pandemic, with protracted gaps in medication fills despite comparatively transient reductions in early healthcare utilization. Our findings are aligned with other studies in non-CKD populations examining care delivery during the pandemic. One recent study using the Medicare Current Beneficiary Survey found approximately 21% of beneficiaries were unable to access care in the early pandemic period, which was varied based on sociodemographic characteristics and comorbidity burden.^21^ For individuals with CKD, however, this may have a differential effect, as early disease management is paramount to reducing risk of adverse clinical outcomes.^22–25^

We further found individuals of younger age, with commercial insurance, Southern US residence, and stage G4 CKD had increased odds of a high care deficit. While not specifically studied, we hypothesize observed trends may be related to differential patterns of pandemic-related misinformation, financial insecurity, medical mistrust, or perceived susceptibility to adverse events among subgroups. Taken together, these findings suggest the acute effects of the pandemic on the CKD population may have substantial downstream effects yet to emerge, and these may be more pronounced in certain subgroups.

CKD is both a risk factor for, and a consequence of, the COVID-19 pandemic. Mounting evidence suggests pre-existing CKD confers worse outcomes for individuals with COVID-19 than for those without pre-existing CKD, including worsened COVID-19 illness severity and increased risk of mortality.^26–29^ Further, as ethnic and racial minorities are disproportionately burdened by CKD, they are also more likely to experience COVID-19 infection, hospitalization, and acute kidney injury (AKI). Therefore, individuals with CKD and COVID-19 infection face a synergistic clinical threat of severe infectious illness, risk of AKI, and death.^18,30–33^ At this time, the additive long-term impact of severe COVID-19 illness among individuals with CKD remains to be seen. Our work builds on this to suggest the burden of the pandemic on the CKD population extends beyond the infection and includes gaps in CKD management. Therefore, the true impact of the COVID-19 pandemic will need to be examined collectively as the summation of direct COVID-19 infection–associated adverse clinical events and disruptions in care independent of infection.

To our knowledge, this is the first study to examine CKD care patterns during the COVID-19 pandemic. Recent work by Patel et al. using Optum Labs data noted a reduction in total outpatient visit volume, yet a twenty-three-fold increase in telemedicine use during the pandemic period compared to the 3 months before the pandemic began.^34^ The study examined these trends across patient demographics, specialties, and diagnoses, and noted variation in the magnitude of utilization patterns while importantly highlighting the detrimental impact of the pandemic across all healthcare segments. Another study noted a reduction in utilization during the early pandemic across six chronic conditions, including diabetes and ESKD.^6^ Absent from both investigations was the evaluation of non-dialysis CKD. Care of earlier CKD is broadly inadequate; the pandemic has worsened that.

The COVID-19 pandemic serves as a natural experiment for the assessment of the value of our care models. Due to rapid policy transformation, reimbursement for telehealth services is no longer as significant a barrier to implementation.^35–37^ We show an early increase in telehealth services, with quick decline as face-to-face encounters were resumed. However, despite an exponential increase in telehealth services, overall encounters (face-to-face plus telehealth) did not restore utilization to pre-pandemic levels. The impacts of this deficit in care for CKD patients will unfold in time. Further, the pandemic provides an opportunity to identify traditional CKD care practices that may be unnecessary and without meaningful impact on outcomes. Detailed evaluation of these relations can inform methods to obtain high-value CKD care.

Notably, provider visits, laboratory assessments, and medication fills all fell substantially relative to expected. Early decreases in care included total clinician visits (about 20%), laboratory assessment for uACR (over 30%), and medication fills (about 8% overall). While a descriptive analysis cannot explain this variance in care delivery, it may be that either patients or professionals perceived laboratory assessment to be less critical to care than provider visits. Also, although the proportion of individuals that had multi-month, automatic, or mailed refills is not available, we hypothesized that these delivery methods would support medication availability during the pandemic. However, our findings revealed a growing decrease in medication refills during the pandemic, suggesting barriers worsened. In a post hoc analysis, we did observe an overall decline in PDC during the pre-pandemic period as well. Therefore, continued pandemic-related PDC reductions, potentially related to appropriate deprescribing in a mature adult cohort, may reflect exacerbation of a trend that began prior to the COVID-19 pandemic.

Our study has limitations. First, our data does not represent all payers, so results may not be fully generalizable. Second, individuals with CKD were identified primarily via medical claims. Due to low levels of CKD diagnoses,^38^ it’s likely we missed many individuals with CKD lacking a diagnosis code, so the effect of COVID on the population with CKD is not fully captured. Third, data from March to August 2021 may not be representative of trends in subsequent pandemic months. Fourth, laboratory testing during the pandemic was likely influenced by a concomitant decline in elective or non-urgent procedures, which was not accounted for in the current analysis. Fifth, we do not have additional data regarding the context of declines in utilization (e.g., provider specialty), laboratory testing, or medication use, which may have been appropriate given the individual circumstance. Finally, our analysis is at a national level and does not account for regional trends in viral outbreaks and state stay-at-home orders.

Despite these limitations, our study has many strengths: a large national CKD cohort with diverse representation, longitudinal evaluation of CKD care trends, and granular evaluation of CKD-relevant management, including all clinician encounters, medications, and lab monitoring. Pharmacy benefit data was available in 96.4% of the population. In sum, individuals with CKD suffered significant disruptions in care utilization, medication fills, and disease monitoring during the early COVID-19 pandemic. The effects of such disruptions on long-term health outcomes and resource utilization remain to be seen. Further, the compounded impact of SARS-CoV-2 infection and observed care disruptions on an at-risk CKD population require intense investigation. Upstream innovation is needed and gaps in care during the pandemic are a call to action to reinvigorate clinical innovations for the stage G3 and G4 CKD populations.

## Supplementary Information

ESM 1 (DOCX 217 kb)

## Data Availability

The data are proprietary and are not available for public use but, under certain conditions, may be made available to editors and their approved auditors under a data-use agreement to confirm the findings of the current study.
