# Supplementary material for: Missing Care: the Initial Impact of the COVID-19 Pandemic on CKD Care Delivery
Source: J Gen Intern Med. 2022 Sep 26;37(16):4241–7. doi: 10.1007/s11606-022-07805-w (PMC9512959; doi:10.1007/s11606-022-07805-w)
Supplement: Supplementary file 1 — (DOCX 217 kb) [file 11606_2022_7805_MOESM1_ESM.docx]

**SUPPLEMENTAL MATERIAL**

**TABLE OF CONTENTS**

**Supplemental Figure S1. Study CONSORT Diagram**

**Supplemental Table S1a. Percent Augmented Care by Telehealth Consultations During Early COVID Period (March 2020-June 2020)**

**Supplemental Table S1b. Percent Augmented Care by Telehealth Consultations During Pre-Vaccine COVID Period (July 2020-Dec 2020)**

**Supplemental Table S1c. Percent Augmented Care by Telehealth Consultations During Late COVID Period (January 2021-August 2021)**

**Supplemental Table S2a. Laboratory Tests with Largest Reduction in PMPM Utilization During the COVID-19 Pandemic**

**Supplemental Table S2b. Reduction in PMPM Utilization of Most Prevalent Laboratory Tests During the COVID-19 Pandemic**

**Supplemental Table S3. Average PDC Difference of Selected AHFS Drug Categories During the COVID-19 Pandemic**

**Supplemental Figure S2. Healthcare Utilization in the Pre-Pandemic Period (January 2018-Feb 2020) by Race**

**Supplemental Table S4. Change in Healthcare Utilization in Pre-Pandemic Period (January 2018-February 2020) to the Pandemic Period (March 2020-August 2021) by Demographics**

**Supplemental Figure S1. Study CONSORT Diagram**

*There are 35,670 members that are found to have CKD based on both claims and labs. The analytical sample contains only the distinct number of members from both claims and labs cohorts.*

Have at least 2 CKD claims at least 90 days apart in 2018 (n = 283,370)

- Medicare (n = 256,254)
- Commercial (n = 27,116)

Have at least 2 <60 eGFR measurements that are at least 90 days apart in 2018 (n = 205,920)

- Medicare (n = 195,262)
- Commercial (n = 10,658)

No ESRD or CKD G5 based on labs and related claims from Jan 2018 to August 2021 (n = 254,983)

- Medicare (n = 229,892)
- Commercial (n = 25,091)

No ESRD or CKD G5 based on labs and related claims from Jan 2018 to August 2021 (n = 193,765)

- Medicare (n = 185,329)
- Commercial (n = 8,436)

Have Stage G3 or G4 ICD 10 codes on the latest claim in 2018 (n = 126,790)

- Medicare (n = 121,087)
- Commercial (n = 5,703)

With latest eGFR lab measurement between >=15 and <60 in 2018 (n = 157,778)

- Medicare (n = 151,423)
- Commercial (n = 6,355)

Analytical sample (n = 248,898)

- Medicare (n = 237,348)
- Commercial (n = 11,550)

With CKD G5 or ESRD based on labs and claims from Jan 2018 to Aug 2021 (n = 28,387)

- Medicare (n = 26,473)
- Commercial (n = 1,914)
- With CKD G5 or ESRD based on labs and claims from Jan 2018 to Aug 2021 (n = 12,155)
- Medicare (n = 11,350)
- Commercial (n = 805)

Excluded

Have Stage G1 or G2 ICD 10 codes on the latest claim in 2018 (n = 128,193)

- Medicare (n = 109,503)
- Commercial (n = 18,690)

With latest eGFR measurement of >=60 (Stage G1 or G2) in 2018 (n = 35,987)

- Medicare (n = 33,906)
- Commercial (n = 2,081)

Excluded

No eGFR measurements (n = 3,168,221)

- Medicare (n = 1,838,800)
- Commercial (n = 1,329,421)

No CKD claims in 2018 (n = 4,952,974)

- Medicare (n = 2,988,020)
- Commercial (n = 1,964,954)

Continuously enrolled from Jan 2018 to August 2021

(n = 5,236,344)

Medicare (n = 3,225,087); Commercial (n = 2,011,257)

No 2 measurements less than 60 at least 90 days apart in 2018 (n = 1,862,203)

- Medicare (n = 1,210,149)
- Commercial (n = 652,054)

Excluded

Excluded

Excluded

Excluded

**Supplemental Table S1a. Percent Augmented Care by Telehealth Consultations During Early COVID Period (March 2020-June 2020)**

| Consultations | Per 1,000 Persons Per Month |
| --- | --- |
| Baseline for the entire cohort | 802.42 |
| Reduction, F2F^[[1]](#footnote-1)^ only (%) | 331.23 (41.28) |
| Reduction, F2F + TH^[[2]](#footnote-2)^ (%) | 158.68 (19.78) |
| Augmented Care (%) | 172.55 (21.50) |

*Note: Results are obtained for combined CKD Stage 3 and 4 members (n = 248,898)*

**Supplemental Table S1b. Percent Augmented Care by Telehealth Consultations During Pre-Vaccine COVID Period (July 2020-Dec 2020)**

| Consultations | Per 1,000 Persons Per Month |
| --- | --- |
| Baseline for the entire cohort | 802.42 |
| Reduction, F2F^[[3]](#footnote-3)^ only (%) | 180.10 (22.44) |
| Reduction, F2F + TH^[[4]](#footnote-4)^ (%) | 55.69 (6.94) |
| Telehealth (%) | 124.41 (15.50) |

*Note: Results are obtained for combined CKD Stage 3 and 4 members (n = 248,898)*

| Supplemental Table S1c. Percent Augmented Care by Telehealth Consultations During Late COVID Period (January 2021-August 2021) Consultations | Per 1,000 Persons Per Month |
| --- | --- |
| Baseline for the entire cohort | 802.42 |
| Reduction, F2F^[[5]](#footnote-5)^ only (%) | 135.83 (16.93) |
| Reduction, F2F + TH^[[6]](#footnote-6)^ (%) | 59.12 (7.37) |
| Telehealth (%) | 76.71 (9.56) |

*Note: Results are obtained for combined CKD Stage 3 and 4 members (n = 248,898)*

**Supplemental Table S2a. Laboratory Tests with Largest Reduction in PMPM Utilization During the COVID-19 Pandemic**

| Laboratory test | Reduction in PMPM utilization (%) | | | | Prevalence (%) |
| --- | --- | --- | --- | --- | --- |
|  | **Overall** | **Early** | **Pre-Vaccine** | **Late** |  |
| Assay of Blood - Carbon Dioxide | -87.68 | -85.05 | -86.94 | -89.55 | 0.81 |
| Metabolic Panel Ionized -Calcium | -85.13 | -88.16 | -84.17 | -84.33 | 2.03 |
| Assay of Serum – Potassium | -75.91 | -74.70 | -74.32 | -77.71 | 4.58 |
| Assay of Blood- Serum Cholesterol | -75.28 | -73.62 | -72.60 | -78.11 | 0.87 |
| Assay of Blood – Chloride | -74.40 | -73.72 | -72.56 | -76.12 | 2.02 |
| General Health Panel | -73.96 | -75.66 | -70.78 | -75.50 | 3.78 |
| Assay of Serum- Sodium | -72.87 | -70.84 | -71.18 | -75.15 | 2.54 |
| Assay of Lipoprotein | -70.09 | -73.14 | -65.47 | -72.04 | 0.67 |
| Assay of Calcium | -69.74 | -68.94 | -67.32 | -71.96 | 2.49 |
| Assay of Triglycerides | -68.59 | -66.20 | -64.91 | -72.55 | 0.83 |

**Supplemental Table S2b. Reduction in PMPM Utilization of Most Prevalent Laboratory Tests During the COVID-19 Pandemic**

| Laboratory Test | Reduction in PMPM utilization (%) | | | | Prevalence (%) |
| --- | --- | --- | --- | --- | --- |
|  | Overall | Early | Pre-Vaccine | Late |  |
| Comprehensive Metabolic Panel | -11.75 | -24.97 | -10.38 | -6.16 | 88.96 |
| Lipid Panel | -18.00 | -29.66 | -16.27 | -13.47 | 83.86 |
| Complete CBC w/ Auto Diff WBC | -12.51 | -25.35 | -10.97 | -7.25 | 83.72 |
| Assay of Thyroid Stim Hormone | -21.62 | -33.62 | -20.13 | -16.74 | 64.75 |
| Urinary Albumin-Creatinine Ratio (Quantitative) | -23.03 | -31.65 | -24.78 | -17.41 | 52.02 |
| Urinary Albumin-Creatinine Ratio (Semi-quantitative) | -24.25 | -31.95 | -25.52 | -19.44 | 48.40 |
| Assay of Urine Creatinine | -24.18 | -32.10 | -25.37 | -19.33 | 47.36 |
| Metabolic Panel Total -Calcium | -38.63 | -45.93 | -35.93 | -37.00 | 46.17 |
| Complete CBC (Automated) | -43.17 | -50.20 | -40.73 | -41.48 | 30.18 |
| Assay of Phosphorus | -38.37 | -42.31 | -36.03 | -38.15 | 18.81 |

**Supplemental Table S3. Average PDC Difference of Selected AHFS**^[[7]](#footnote-7)^* **Drug Categories During the COVID-19 Pandemic**

| AHFS Category |  | PDC^[[8]](#footnote-8)^ Difference  (Variable Denominator) | | | | Prevalence (%) |
| --- | --- | --- | --- | --- | --- | --- |
|  | Pre-Baseline | Overall | Early | Pre-Vaccine | Late |  |
| Thiazide Diuretics | -0.18 | -0.20 | -0.12 | -0.19 | -0.26 | 12.90 |
| Angiotensin-Converting Enzyme Inhibitors | -0.13 | -0.14 | -0.08 | -0.13 | -0.18 | 29.07 |
| Angiotensin II Receptor Antagonists | -0.24 | -0.14 | -0.09 | -0.13 | -0.17 | 32.68 |
| Sulfonylureas | -0.12 | -0.14 | -0.08 | -0.12 | -0.18 | 15.18 |
| Proton Pump Inhibitors | -0.14 | -0.14 | -0.09 | -0.13 | -0.17 | 29.07 |
| Biguanides | -0.13 | -0.14 | -0.08 | -0.12 | -0.18 | 19.95 |
| Loop Diuretics | -0.11 | -0.13 | -0.08 | -0.12 | -0.15 | 25.14 |
| Insulins | -0.09 | -0.12 | -0.08 | -0.12 | -0.13 | 10.25 |
| Beta Adrenergic Blocking Agents | -0.09 | -0.10 | -0.06 | -0.10 | -0.13 | 49.85 |
| Opiate Agonists | -0.08 | -0.09 | -0.07 | -0.09 | -0.11 | 22.55 |
| Antigout Agents | -0.07 | -0.09 | -0.05 | -0.09 | -0.10 | 13.82 |

**Supplemental Figure S2. Healthcare Utilization in the Pre-Pandemic Period (January 2018-Feb 2020) by Race**

**
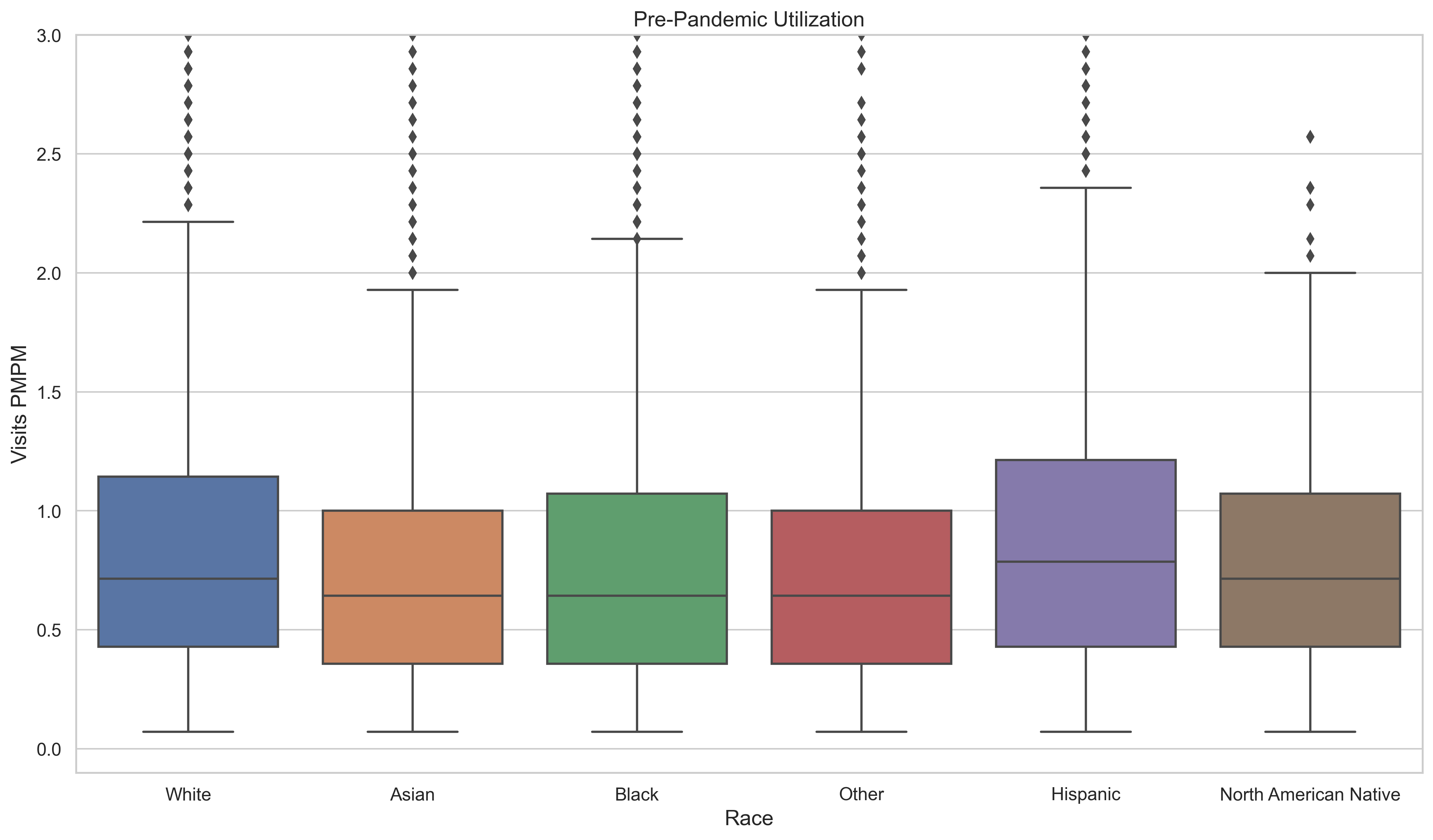
**

**Supplemental Table S4. Change in Healthcare Utilization in Pre-Pandemic Period (January 2018-February 2020) to the Pandemic Period (March 2020-August 2021) by Demographics**

|  |  | **Count** | **Mean (SD)** | **Median [p25, p75]** | **[Min, Max]** |
| --- | --- | --- | --- | --- | --- |
| Age Group | 18-44 | 134 | 0.39 (0.97) | 0.38 [-0.15, 0.90] | [-2.76, 3.30] |
|  | 45-54 | 832 | 0.34 (0.91) | 0.30 [-0.17, 0.83] | [-3.79, 4.02] |
|  | 55-64 | 5,671 | 0.32 (0.87) | 0.26 [-0.17, 0.79] | [-11.13, 7.40] |
|  | 65-74 | 52,496 | 0.16 (0.78) | 0.14 [-0.26, 0.57] | [-6.58, 12.25] |
|  | 75-84 | 98,417 | 0.17 (0.78) | 0.16 [-0.25, 0.60] | [-8.54, 7.94] |
|  | 85+ | 62,325 | 0.15 (0.77) | 0.15 [-0.25, 0.57] | [-7.12, 12.31] |
| Sex | Male | 89,379 | 0.19 (0.78) | 0.17 [-0.24, 0.61] | [-11.13, 12.25] |
| Race | White | 168,246 | 0.18 (0.79) | 0.17 [-0.25, 0.60] | [-8.54, 12.31] |
|  | Asian | 3,365 | 0.12 (0.73) | 0.10 [-0.28, 0.50] | [-5.51, 3.65] |
|  | Black | 39,429 | 0.12 (0.76) | 0.12 [-0.29, 0.53] | [-11.13, 5.40] |
|  | Hispanic | 4,213 | 0.25 (0.82) | 0.22 [-0.20, 0.69] | [-4.52, 7.40] |
|  | Native American | 279 | 0.23 (0.73) | 0.21 [-0.15, 0.60] | [-2.10, 3.50] |
|  | Other | 4,343 | 0.11 (0.72) | 0.10 [-0.27, 0.48] | [-4.68, 4.00] |
| Insurance Type | Commercial | 915 | 0.34 (0.82) | 0.29 [-0.11, 0.77] | [-2.27, 4.71] |
|  | Medicare | 218,960 | 0.17 (0.78) | 0.15 [-0.25, 0.59] | [-11.13, 12.31] |
| USR Class^[[9]](#footnote-9)^* | Urban | 54,327 | 0.16 (0.78) | 0.15 [-0.25, 0.58] | [-6.97, 12.25] |
|  | Rural | 95,248 | 0.15 (0.77) | 0.14 [-0.26, 0.57] | [-11.13, 8.65] |
|  | Suburban | 70,120 | 0.19 (0.80) | 0.17 [-0.24, 0.62] | [-8.54, 12.31] |
| Geographic Region | Northeast | 31,884 | 0.15 (0.78) | 0.13 [-0.27, 0.56] | [-7.12, 7.94] |
|  | Midwest | 30,588 | 0.06 (0.74) | 0.08 [-0.33, 0.47] | [-6.50, 4.21] |
|  | South | 118,238 | 0.19 (0.79) | 0.17 [-0.23, 0.62] | [-11.13, 12.31] |
|  | West | 39,160 | 0.19 (0.78) | 0.16 [-0.23, 0.61] | [-6.58, 7.30] |
| CKD Stage | 3 | 207,075 | 0.16 (0.78) | 0.15 [-0.25, 0.59] | [-11.13, 12.31] |
|  | 4 | 12,800 | 0.23 (0.78) | 0.21 [-0.19, 0.66] | [-5.09, 7.30] |

1. ^F2F: Face-to-face^ [↑](#footnote-ref-1)
2. ^TH: Telehealth^ [↑](#footnote-ref-2)
3. ^F2F: Face-to-face^ [↑](#footnote-ref-3)
4. ^TH: Telehealth^ [↑](#footnote-ref-4)
5. ^F2F: Face-to-face^ [↑](#footnote-ref-5)
6. ^TH: Telehealth^ [↑](#footnote-ref-6)
7. * AHFS: American Hospital Formulary Service [↑](#footnote-ref-7)
8. ^PDC: Proportion of days covered^ [↑](#footnote-ref-8)
9. * USR: Urban-suburban-rural [↑](#footnote-ref-9)
